# *Rhodostrophia crypta*, a new species from Middle Asia (Lepidoptera: Geometridae)

**DOI:** 10.3897/BDJ.8.e52462

**Published:** 2020-05-04

**Authors:** Jaan Viidalepp, Igor Kostjuk

**Affiliations:** 1 Estonian University of Life Sciences, Tartu, Estonia Estonian University of Life Sciences Tartu Estonia; 2 Zoological Museum, Kyiv National Taras Shevchenko Universit, Kiev, Ukraine Zoological Museum, Kyiv National Taras Shevchenko Universit Kiev Ukraine

**Keywords:** Taxonomy, morphology, new species, Kazakhstan, Turkmenistan

## Abstract

**Background:**

*Rhodostrophia* is a speciose genus which is widespread in arid landscapes of Central Asia.

**New information:**

A new species, *Rhodostrophia crypta* sp. n. is described below from Kazakhstan.

## Introduction

Hugo Christoph (Christoph 1877, Christoph 1885) collected a series of moths in the vicinity of Krasnovodsk (now Turkmenbashi) on the Turkmen shore of the Caspian Sea and supplemented his original description of *Rhodostrophia vastaria* Christoph with line illustrations of a male and a female moth. Uvarov (1910) referred to *R. vastaria* from the South Urals.

*R. vastaria* was recorded as occurring in the territories of the Turkmen and Kazakh Soviet Republics in check lists by Viidalepp (1976), Viidalepp (1988) and Hausmann (2004), with a figure of male genitalia. However, a recent search for *R*. *vastaria* types in the collection of the ZISP, St. Petersburg has yielded information about two female specimens (Trusch and Hausmann 2007). New localities for *R. vastaria* in western Kazakhstan were listed by Gorbunov (2011). The species is repeatedly collected on the Ustjurt plateau, some hundreds of kilometres north of the type locality of *R. vastaria*.

## Materials and methods

New '*Rhodostrophia vastaria* Christoph' records come from eastern Kazakhstan, specifically in the vicinity of Balkhash lake where the large lepidopterological collection of Dr A. Pototski was deposited in the IZBE insect collection (now hosted at the Estonian University of Life Sciences). The distance between western collecting sites and new localities near Lake Balkhash is quite large. Authentic lectotype data for R. vastaria were published by Trusch & Hausmann (2007) and some material of *Rhodostrophia vastaria* for dissection was found in the Museum of Zoology of the Kyiv National Taras Shevchenko University.

Detailed comparison of moths from eastern and western populations of putative *R. vastaria* yields differences both in the external appearance of adults and in the build of their genitalia. We describe the eastern Kazakh populations as *Rhodostrophia crypta*, sp. n. below. "Crypta", as a noun, is a derivative from "cryptic" ~hidden.

Prout (1912) referred to the similarity of these moths with *Rhodostrophia jacularia *Hübner, 1813 and *R. badiaria* Freyer, 1841. Sterneck (1941) commented on the small size of the male genitalia of *R. vastaria* compared to those of* R. jacularia* Hübner.

Material. *Rhodostrophia vastaria* Christoph: Digital images of the female lectotype and the corresponding genitalia slide. Digital images of moths and their genitalia slides from Ustjurt plateau (western Kazakhstan) from D. Shovkoon.

Additional material. A paper photo of a male from "Ili" in the collection of ZFMK (Bonn).

### External characteristics of *Rhodostrophia vastaria* Christoph and the new species *R. crypta*

The genus *Rhodostrophia* is characterised by their quadripectinate male antennae (i.e. there are two pairs of long rami on each antennomere), by the presence of two accessory cells in the forewing venation. All the species of *Rhodostrophia* have the number of their hind tibial spurs reduced, with exception of *R. jacularia* Hübner, *R. vastaria, R. tabestana *Trusch& Hausmann and the new species *Rhodostrophia crypta* sp. n.with four fully-developed spurs on their hind legs. Wings of R. *vastaria* and *R. crypta*, sp. n. are scaled yellowish-grey, forewings with fragmented postmedial and antemedial fasciae.

### The characteristics of *Rhodostrophia vastaria* Christoph, 1877

The antemedial fascia of the forewing is not straight but shifted downwards on the hind margin of the forewing. The hind wing postmedial fascia is smooth, not projecting outwards at the vein M3. The distal end of a valva is concave at the middle and bulged ventrally. The forked sclerite in the bursa copulatrix of the female is large.

## Data resources

Materials from the collections of the Estonian University of Life Sciences (the IZBE (Institute of Zoology and Botany of Estonian Academy of Sciences) collection, Tartu, Estonia) and the Lepidoptera collection of the Zoological Museum in Kyiv National Taras Shevchenko University and Zoologischer Staatssammlung München were studied.

## Taxon treatments

### Rhodostrophia crypta

Viidalepp & Kostjuk 2020 sp. n.

E76CB88A-1C84-5D7E-9744-DED73ACE9083

#### Materials

- **Type status:** Holotype. **Occurrence:** recordedBy: A. Pototski; U. Jürivete; individualCount: 1; sex: male; otherCatalogNumbers: IZBE3026002; **Taxon:** order: Lepidoptera; family: Geometridae; genus: Rhodostrophia; specificEpithet: crypta; taxonRank: species; **Location:** continent: Asia; country: Kazakhstan; locality: NW of Uch-Aral; verbatimElevation: 400 m; decimalLatitude: 46.39666667; decimalLongitude: 80.71555556; **Identification:** identifiedBy: Jaan Viidalepp; Igor Kostjuk; **Event:** samplingProtocol: at light; eventDate: 21-5-2004; year: 2004; month: 5; day: 21; **Record Level:** type: Physical object; institutionID: Estonian University of Life Sciences, Entomological Collection ; collectionCode: IZBE; basisOfRecord: Preserved specimen
- **Type status:** Paratype. **Occurrence:** recordedBy: Danilevsky; individualCount: 1; sex: female; **Taxon:** order: Lepidoptera; family: Geometridae; genus: Rhodostrophia; specificEpithet: crypta; taxonRank: specie; **Location:** continent: Asia; country: Kazakhstan; locality: Khantau 800 m, Balkhash-See; **Identification:** identifiedBy: Jaan Viidalepp; Igor Kostjuk; **Event:** samplingProtocol: at light; eventDate: 12-5-1991; year: 2014; month: 6; day: 1; **Record Level:** type: Physical object; institutionID: Museum of Zoology, Kyiv National Taras Shevchenko University; collectionCode: ZMKU; basisOfRecord: Preserved specimen
- **Type status:** Paratype. **Occurrence:** recordedBy: A. Pototski; U. Jürivete; individualCount: 1; sex: male; otherCatalogNumbers: IZBE3026001; **Taxon:** order: Lepidoptera; family: Geometridae; genus: Rhodostrophia; specificEpithet: crypta; taxonRank: species; **Location:** continent: Asia; country: Kazakhstan; locality: Charyn valley; verbatimElevation: 1200 m; **Identification:** identificationID: BarcodeZSM Lep 54333; identifiedBy: Jaan Viidalepp; Igor Kostjuk; identificationQualifier: identified by dissection and barcoding; **Event:** eventID: collecting at light; samplingProtocol: at light; eventDate: 1-6-2014; year: 2014; month: 6; day: 1; **Record Level:** type: Physical object; institutionID: Estonian University of Life Sciences; collectionCode: IZBE; basisOfRecord: Preserved specimen
- **Type status:** Paratype. **Occurrence:** recordedBy: A. Pototski; U. Jürivete; individualCount: 1; sex: male; otherCatalogNumbers: IZBE3026000; **Taxon:** order: Lepidoptera; family: Geometridae; genus: Rhodostrophia; specificEpithet: crypta; taxonRank: species; **Location:** continent: Asia; country: Kazakhstan; locality: Charyn vally; verbatimElevation: 1200 m; **Identification:** identifiedBy: Jaan Viidalepp; Igor Kostjuk; **Event:** samplingProtocol: at light; eventDate: 1-6-2014; year: 2014; month: 6; **Record Level:** type: Physical object; institutionID: Estonian University of Life Sciences; collectionCode: IZBE; basisOfRecord: Preserved specimen
- **Type status:** Paratype. **Occurrence:** recordedBy: R. Yakovlev; individualCount: 2; sex: female; otherCatalogNumbers: BC ZSM Lep 54333; **Taxon:** order: Lepidoptera; family: Geometridae; genus: Rhodostrophia; specificEpithet: crypta; taxonRank: species; **Location:** continent: Asia; country: Kazakhstan; locality: Tarbagatai distictr.; Zhagalbaily Mts; **Identification:** identifiedBy: Axel Hausmann, Jaan Viidalepp, Igor Kostjuk; **Event:** samplingProtocol: at light; eventDate: 18-6-2014; year: 2014; month: 6; day: 18; **Record Level:** type: Physical object; institutionID: Zoologisches Staatssammlung München; collectionCode: ZSM; basisOfRecord: Preserved specimen

#### Description

Sandy yellowish-grey moths with wing span 25–26 mm. Dark irroration stronger on wings, forewing postmedial fascia broader at costa; wing markings are less reduced than in R. vastaria. Underside of wings almost monotonous, greyish.

#### Taxon discussion

*Rhodostrophia vastaria* and *Rhodostrophia crypta*, sp. n. are superficially similar but differing in characteristics of male and female genitalia, as discussed below.

## Discussion

A differential diagnosis of ***Rhodostrophia crypta*, sp. n.**:

Wing span of moths 25–26 mm (Fig. 1a, b). The Uch-Aral male is grey with a conspicuous dark grey pattern and dusting (Fig. 1a); its hindwing postmedial line is outwardly dentate at the vein CuA1 and the forewing medial area seems relatively broader. West Kazakh moths (Fig. 1c, d) of *R. vastaria* are evenly sand-coloured, yellowish-grey and with sparse grey maculation. The sandy grey ground colour of the moths from the Balkhash region is more intensively covered by brown spots and the postmedial line is more suffused on hindwings (Fig. 1a, b).

The distal edge of the valva in the male genitalia is roundly bulged at the saccular corner in *R. vastaria *(Fig. 2a; Hausmann 2004: Fig. 174b), but it is straight in *R. crypta*, sp. n. (Fig. 2b). Female genitalia of moths are also different; moths of western population have the seventh segment of the abdomen and the tubular sclerotisation of the ductus bursae distinctly longer in moths of the western population (Fig. 3a) and the forked sclerite in the corpus bursae is also larger in western moths than in those moths from the eastern Kazakh population (Fig. 3b).

The differences in male and female genitalia structures and wing pattern between the western and eastern Kazakh populations justify the separation of the Balkhash lake shore populations as *Rhodostrophia crypta* Viidalepp & Kostjuk, sp. n.

Similar species. *Rhodostrophia jacularia* has a very different, clear and contrasting wing pattern but similar male genitalia (with the distal margin of the valva smoothly rounded) and *R*. *tabestana* (Trusch and Hausmann 2007) has quite similar wings and colouration, but the distal margins of valvae are not straight; rather they are slightly concave. Genetically nearest species: *Rhodostrophia jacularia* Hübner (3.7%). The distally truncate shape of valva and the presence of a cornutus on the vesica in *R*. *jacularia*,* R. crypta*, sp. n.,* R. vastaria* and *R. tabestana* allow them to be combined together in the *Rhodostrophia jacularia* species group.

### Ecology

The moths of the new species were collected in steppe landscapes.

### Distribution of *Rhodostrophia vastaria* and *R. crypta,* sp. n.

**Figure 4.**
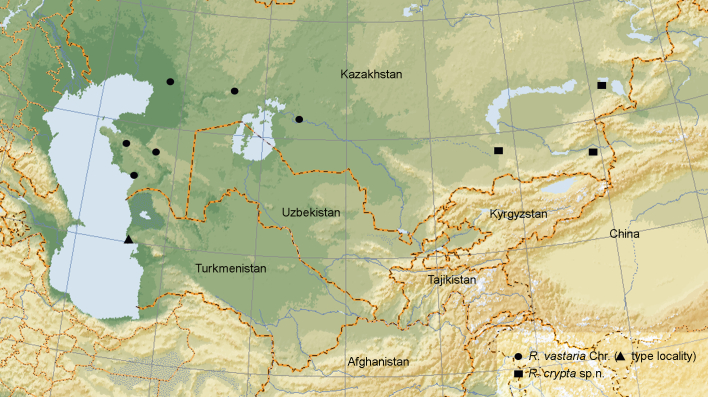
Distribution map of *R. vastaria* and *R. crypta*, sp. n.

The distribution area of *Rhodostrophia vastaria* is fragmented between Turkmenbashi in Turkmenistan, the Ustjurt plateau in western Kazakhstan and the southern Urals (Fig. 4). *Rhodostrophia crypta*, sp. n. is an eastern Kazakh species. Both species do not appear in the recent review of Chinese *Rhodostrophia* (Cui et al. 2019).

## Supplementary Material

XML Treatment for Rhodostrophia crypta

## Figures and Tables

**Figure 1a.**
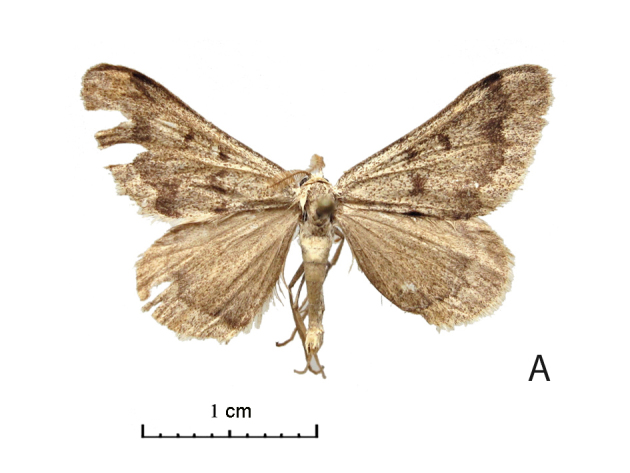
*Rhodostrophia crypta*, sp. n. (Holotype, male, IZBE)

**Figure 1b.**
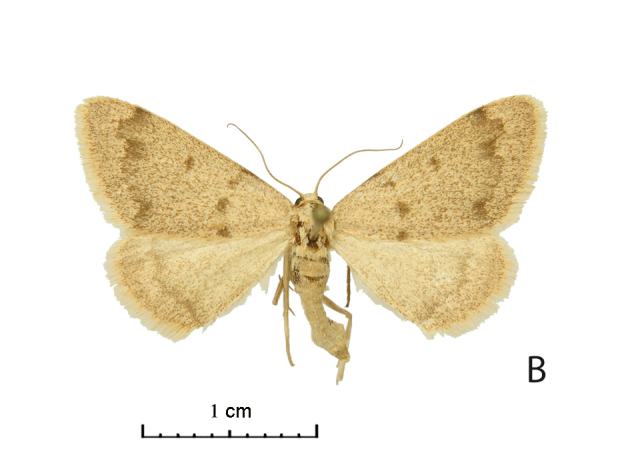
*Rhodostrophia crypta*, sp. n. (Paratype, female, ZMKU)

**Figure 1c.**
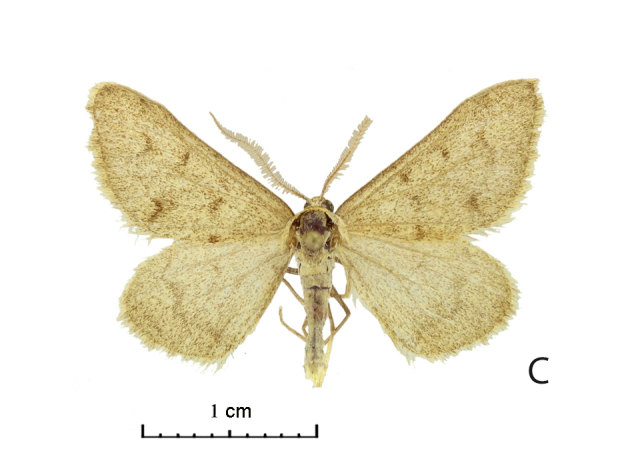
*Rhodostrophia  vastaria *Christoph, male, Ustjurt plateau, Sai-Utjos, Shovkoon leg.

**Figure 1d.**
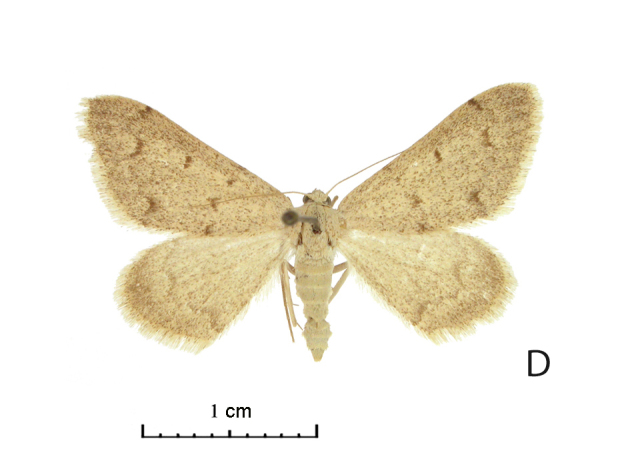
*Rhodostrophia  vastaria* Christoph (Lectotype, female, ZISP)

**Figure 2a.**
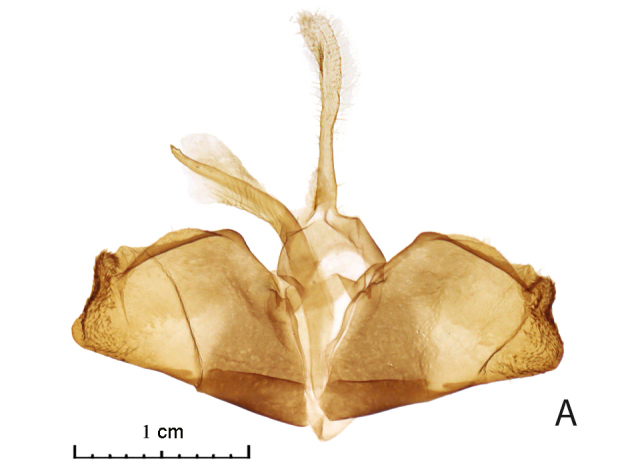
*Rhodostrophia* *vastaria*, male, Ustyurt plateau, slide 114, Shovkoon

**Figure 2b.**
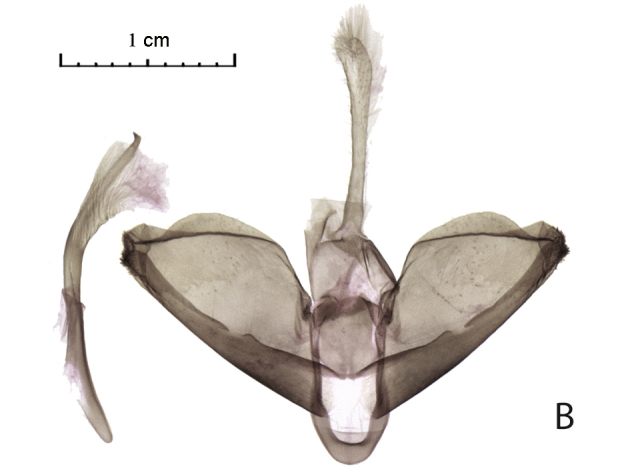
*Rhodostrophia* *crypta*, sp. n. (Paratype, male, slide 8976 IZBE)

**Figure 3a.**
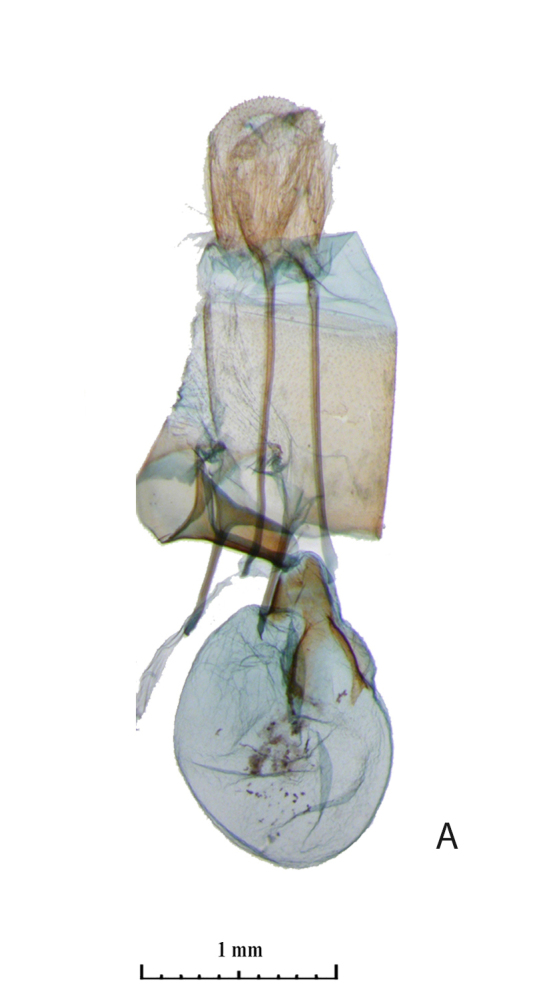
*Rhodostrophia* *vastaria *(Lectotype, female, gen. prep. 634/2005 Trusch, ZISP)

**Figure 3b.**
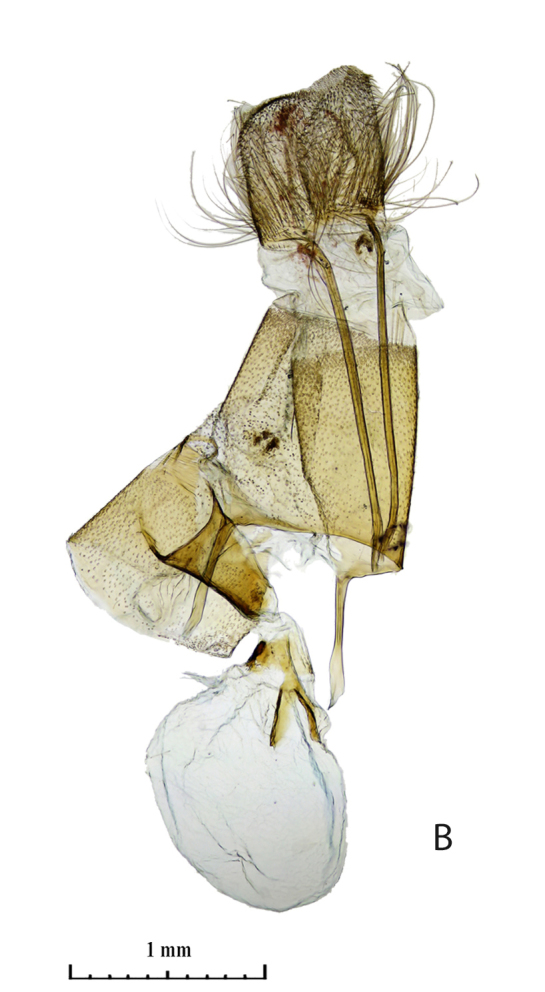
*Rhodostrophia* *crypta*, sp. n. (Paratype, female slide 488, ZMKU)

## References

[B5572588] Christoph H. (1877). Sammelergebnisse aus Nordpersien, Krasnowodsk in Turkmenien und dem Daghestan. Horae Societatis Entomologicae Rossicae.

[B5572607] Christoph H., Romanoff N. M. (1885). Lepidoptera aus dem Achal-Tekke Gebiet. Zweiter Teil. Memoires sur les lepidopteres par N.M. Romanoff, Tome 2.

[B5572640] Cui Le, Xue Dayong, Jiang Nan (2019). Description of two new species of *Rhodostrophia* Hübner, 1823 from China (Lepidoptera, Geometridae). Zootaxa.

[B5572622] Gorbunov P J (2011). Higher moths (Macrolepidoptera) of deserts and southern steppes of Western Kazakhstan. Review of the fauna..

[B5572631] Hausmann A. (2004). Geometrid moths of Europe. Volume 2. Sterrhinae..

[B5572650] Prout L. B. (1912). Die Spanner des Palaearktischen Faunengebietes. Die Gross-Schmetterlinge der Erde, Volume 4..

[B5572662] Sterneck J (1941). Versuch einer Darstellung der systematischen Beziehungen bei den Palearktischen Sterrhinae (Acidaliinae). Studien über Sterrhinae (Acidaliinae) IX. Die Gattung Rhodostrophia und deren nahe Verwandten.. Zeitschrift des Wiener Entomologischen Vereines, Jg. 26.

[B5572672] Trusch R., Hausmann A. (2007). A new species of the genus *Rhodostrophia* Hübner, 1823 from Iran (Geometridae: Sterrhinae).. Nota lepidopterologica.

[B5572682] Uvarov B. P. (1910). To lepidopterofauna of transural Kirghis steppes.. Revue Russe d’Entomologie.

[B5572692] Viidalepp J. (1976). Checklist of the Geometridae of the fauna of the USSR. I.. Entomologicheskoe Obozrenie.

[B5572702] Viidalepp J. (1988). Geometrid moths of mountainous Middle Asia..

